# Realist review of nature-based interventions for men: understanding the contexts and mechanisms necessary for successful outcomes

**DOI:** 10.1186/s12889-026-26867-7

**Published:** 2026-03-05

**Authors:** Josh Dumbrell, W Masterton, H Carver, T Parkes

**Affiliations:** Salvation Army Centre for Addiction Services and Research, Faculty of Health, Sport and Society, University of Stirling, Stirling, UK

**Keywords:** Greenspace, Nature-based interventions, Men’s wellbeing, Realist review, Masculine identity, Social inclusion, Health disparities, Community-based programmes, Gender-responsive interventions, Peer-led support

## Abstract

**Background:**

Men face significant health disparities, including higher rates of premature death, mental health challenges, and substance use disorders. Nature-based interventions hold promise for improving men’s health and wellbeing by leveraging natural environments and structured activities aligned with men’s preferences for practical, action-oriented solutions. This realist review explores the contexts, mechanisms, and outcomes of nature-based interventions, providing a gender-specific perspective on how these interventions benefit men.

**Methods:**

A realist synthesis was conducted following Pawson et al.’s iterative framework and the RAMESES reporting standards. Initial programme theories were constructed through early interrogation of the men’s health and greenspace literatures, and expert consultations. Data extraction focused on identifying context-mechanism-outcome configurations to refine programme theories. Analysis involved synthesising findings from qualitative, quantitative, and grey literature to demonstrate how nature-based interventions influence men’s wellbeing.

**Results:**

The review identified nine refined programme theories, encompassing three domains: *Being*, *Doing*, and *Growing Together*. Calming natural settings (PT1) and tailored physical activities (PT2) reduced stress, improved fitness, and enhanced resilience. Purposeful engagement (PT3) and skill development in peer-led, supportive environments (PT4) fostered self-efficacy, identity reformation, and empowerment. Strengths-based, inclusive approaches (PT5, PT6) built social bonds and community cohesion, while structured cognitive engagement (PT7) enhanced problem-solving and collaborative skills. Foundational contexts, including safe, non-judgemental, culturally sensitive spaces (PT8) and tailored, multidisciplinary support (PT9), underpinned these mechanisms, enabling sustained health and wellbeing outcomes.

**Conclusions:**

Nature-based interventions offer a powerful and gender-responsive solution to men’s mental health challenges, combining restorative natural environments, purposeful activities, and peer-led support. Tailored interventions that align with men’s cultural identities and values significantly enhance engagement, self-efficacy, and social cohesion. These findings provide actionable insights for designing inclusive, impactful nature-based interventions, addressing longstanding health disparities. Future research should explore adapting nature-based interventions to diverse populations and contexts to maximise their reach and effectiveness.

**Protocol registration:**

CRD42023487594

**Supplementary Information:**

The online version contains supplementary material available at 10.1186/s12889-026-26867-7.

## Background

International evidence highlights significant health disparities between men and women, with men facing higher rates of premature death and chronic illnesses such as heart disease, certain mental health issues, suicide, and substance use disorders [1–4]. Men are also more likely to exhibit externalising disorders – often called disruptive behaviour disorders – including attention deficit hyperactivity disorder, oppositional defiant disorder, and conduct disorder, while they engage in risky behaviours like smoking and substance use at higher rates, and are less inclined to seek healthcare support [5–8]. They also disproportionately face social challenges such as homelessness and incarceration, making up 93% of the global prison population and about 70% of those experiencing homelessness in the UK, US, and Canada [9–14]. Despite a few national efforts to implement gender-informed health strategies, the issue of men’s health continues to be relatively neglected in academic and political fora, compared to women’s health [3, 4, 8, 15–18]. Recent global policy analyses echo this pattern: the Lancet Commission on gender and global health shows that most gender-equality work still frames gender almost exclusively around women and girls, with only 3% of surveyed organisations explicitly recognising men and boys as beneficiaries of gender equality efforts [19]. The World Health Organisation’s (WHO) [2] *Strategy on the health and well-being of men in the WHO European Region* is a noted exception, advocating for the prioritisation of gender equality, the creation of gender-responsive health systems, and enhanced health promotion.

Among their broad portfolio, the WHO [2] acknowledge the potential for greenspace (natural or vegetated areas accessible for recreation and relaxation), and nature-based interventions (NBIs) (therapeutic activities using nature) to enhance human physical and mental health [20, 21]. A meta-analysis of 143 studies found that greenspace exposure significantly reduces diastolic blood pressure, salivary cortisol, heart rate, diabetes incidence, all-cause mortality, and cardiovascular mortality among women and men [22]. Another systematic review reported that 90% of the included studies found positive effects of nature-based recreation on mental health including mood, cognitive function, and reductions in anxiety and depressive symptoms [23].

NBIs may be especially relevant for men experiencing health problems, substance use and social challenges, such as unemployment or homelessness, who may be among the most disconnected from supportive environments. Recent work on nature- and place-based interventions suggests that NBIs work best when they reconnect people to local green and blue spaces in ways that reflect local histories, cultures, and inequalities [24]. Many men report preferring the company of other men [25], and are drawn to outdoor physical activities that use practical skills and problem-solving (e.g., orienteering, camping) [26–30]. Cross-disciplinary evidence showing that men often endorse ‘practical solutions’ and ‘problem solving’ for managing stress and depression [31]. This is consistent with broader norms that discourage emotional disclosure which may foster a preference for more cognitive–behavioural approaches over traditional talking therapies [32]. Given these distinct preferences, unmet needs, and the clear potential of NBIs, a comprehensive review of effective interventions for men is an essential first step toward addressing such health disparities.

Employing a realist methodology offers a robust analytical framework, ideal for examining complex interventions like NBIs. Realist review (or synthesis) permits an understanding of causation in complex settings by identifying the relationships between contexts and mechanisms and how these lead to outcomes [33]. More specifically, this approach views causation as contexts (C) and activating mechanisms (M) that produce outcomes (O). This is a more complex and context-sensitive process than linear causation which is typically consistent with tightly controlled experiments within the natural sciences. This interplay of context, mechanisms, and outcomes is known as a CMO configuration (CMOC) and is commonly expressed as an ‘if-then-because’ statement, explaining not only *what* happens, but *why*, in specific situations [34, 35].

A realist critique of the Cochrane approach to reviewing complex social programmes in public health argues that the method of aggregating data using counterfactual reasoning from experimental trials often fails to capture the complexities inherent in socially contingent public health programmes [36]. Contrastingly, a realist approach accommodates a broad range of evidence types, including qualitative data and expert opinion, which are often excluded from traditional systematic reviews [33, 35]. Such flexibility enables richer understanding, substantiates evidentiary blank spots [33, 35], and can help to build or refine theoretical frameworks, especially in conceptual areas where understanding is limited such as NBIs for men.

Realist review methods are increasingly used in the synthesis of complex public health interventions [33], which, by nature, involve multiple components and feedback mechanisms [37]. Greenspace interventions, for example, operate in dynamic settings and involve interdisciplinary teams, with outcomes shaped by contextual factors [38]. NBIs also fit this complexity given that they encompass diverse activities like physical exercise and mindfulness which interact with participants’ psychological states and the context in which they are delivered [39, 40]. Context can heavily influence outcomes; for instance, care farming may be perceived differently if mandated punitively rather than as rehabilitation [41].

Realist methodologies are thus ideal for unpacking the complexity of NBIs. They reveal how contextual factors (C), intervention components, and mechanisms – comprising mechanism resources (e.g., activities, support structures) and mechanism reasoning (e.g., motivation, cognitive processes) – interact to shape outcomes [42]. Given the diversity of NBIs and varied outcomes, realist methods offer a theory-driven approach to explain why certain results occur, with or without quantitative data [40, 43, 44]. This approach highlights how contexts like study design and population influence engagement and outcomes [45], making it essential for understanding the nuances of NBIs.

Masterton and colleagues (2020) [38] provided a foundational framework for the present examination of men’s health and greenspace literature. The authors’ gender-neutral realist review of greenspace interventions, identified three themes: ‘nature’, ‘individual self’, and ‘social self’. Under ‘nature’, personal and sensory connections to greenspace promoted relaxation and self-reflection, reducing stress and fostering readiness for change. The ‘individual self’ theme involved supportive programmes with experienced facilitators that build skills and confidence, while structured routines enhanced self-esteem and identity transformation. The ‘social self’ theme encompassed supportive relationships and a collaborative culture that encouraged social engagement, improved interpersonal skills, and sustained recovery support. These themes link context to mechanisms – resources like skilled facilitators and activities, and reasoning such as building self-efficacy and a sense of purpose – to shape positive outcomes. These mechanisms are particularly relevant for men given they have been shown to enhance resilience and positively address self-harming behaviours and suicidal ideation [31, 46].

Pawson et al. (2005) [33] outline a five-step, iterative process for conducting realist reviews, allowing for the refinement of programme theories as evidence emerges. This flexible framework supports the development and understanding of complex interventions like NBIs, accommodating diverse evidence types and addressing gaps in knowledge [33, 35].

### Aims and objectives of the review

The aim of this review was to determine the underlying mechanisms which occur within specific contexts, and how these lead to desired outcomes in NBIs for men’s health and wellbeing. Wellbeing (or well-being) is defined as the combination of experiencing positive emotions, such as happiness and contentment, and ‘functioning effectively’ in the world. This includes maintaining positive relationships, having control over one’s life, and possessing a sense of purpose [47]. The review aimed to answer the following research questions (RQs):

1. What components and activities are incorporated in NBIs that aim to enhance the health and wellbeing of men?
2. What outcomes (O) are associated with these NBIs for men?
3. What are the underlying mechanisms (M) that facilitate the emergence of these outcomes in NBIs for men?
4. In what contexts (C) do these mechanisms operate to produce the observed outcomes in NBIs for men?
5. What configurations of context, mechanism, and outcome (C-M-O) can explain the impact of NBIs on men’s health and wellbeing, in different population groups.

## Methods

This review followed Pawson et al.’s (2005) [33] strategy for realist synthesis, as summarised immediately below, and is reported in line with the publication standards outlined by Wong et al. (2013) [35].

Key steps in realist review [33]:

i. Clarify scope and theory – define review questions, purpose, and initial programme theories.
ii. Search iteratively – conduct exploratory, then progressively-focused, theory-driven searches.
iii. Appraise and extract – judge relevance/rigour and extract bespoke CMO data from each study.
iv. Synthesise and refine – configure evidence to refine programme theories (what works, for whom, how, in what contexts).
v. Disseminate and apply – turn refined theories into context-sensitive recommendations and implementation guidance.

### Protocol registration

A protocol for this realist review was developed and registered with the International Prospective Register of Systematic Reviews (PROSPERO) [48], under registration number CRD42023487594. This ensured that the review adhered to established guidelines for systematic review reporting and maintained methodological transparency.

### Formation of initial programme theories

Initial programme theories (IPTs) are hypotheses about how interventions work in specific contexts, and these hypotheses are then tested and refined throughout the review. IPTs were developed to explore how NBIs impact men’s health and wellbeing, focusing on identifying causal mechanisms and the contexts in which they occur using the CMOC. These theories were formulated through exploratory reading, stakeholder discussions, and expert consultations, then refined by systematically searching and extracting data from the literature. Masterton et al.‘s (2020) [38] framework was drawn on to assess contexts, mechanisms, and outcomes specific to men in NBIs, ensuring the framework was continuously reviewed and modified to reflect these elements accurately. The IPT table is shown in Table 1.

**Table 1:** Initial programme theories identified to be tested and refined

Programme theory #	Contexts	Mechanisms	Outcomes
Nature-based interventions: Being			
1: Therapeutic nature environment	• Restorative natural settings • Variety in natural landscapes • Easy accessibility to nature • Programming supporting solitude	• Experience of calmness and relaxation • Activation of parasympathetic response • Soft fascination with nature • Self-reflection and introspection	• Reduced stress • Improved mental wellbeing • Enhanced recovery from mental fatigue • Enhanced personal growth and stress reduction
If NBIs offer restorative settings with diverse landscapes and easy access to nature, supported by programming that allows solitude (context), then participants will experience calmness, relaxation, and activation of their parasympathetic response, engaging in soft fascination and self-reflection (mechanism), because this leads to reduced stress, enhanced recovery from mental fatigue, improved mental wellbeing, and personal growth (outcome).			
2: Engaging with nature’s diversity	• Biodiversity and rich natural environments • Sensory engagement with nature • Inclusion of various nature-based activities	• Indirect attention • Perceived connection with nature • Sense of agency	• Increased wellbeing • Feeling ‘right sized’ • Stabilised mood
If participants engage with biodiverse, rich natural environments through sensory experiences and varied nature-based activities (context), then they will feel a connection with nature and experience a sense of agency through indirect attention (mechanism), because this promotes stabilised moods, improved wellbeing, and a feeling of being ‘right-sized’ (outcome).			
Physical activity and wellbeing: Doing			
3: Physical activities in programmes	• Availability of diverse physical activities • Inclusion of structured activities appealing to men • Balance between group activities and individual challenges in nature	• Enjoyment and interest • Physical empowerment • Sense of agency • Identity formation	• Increased physical fitness • Enhanced mental health • Greater overall engagement in the programme • (Re)connection with (masculine) self
If NBIs offer diverse, structured physical activities that balance individual challenges and group tasks appealing to men (context), then participants will enjoy the activities, feel physically empowered, and re-form their masculine identities (mechanism), because this leads to increased physical fitness, enhanced mental health, and greater engagement in the programme (outcome).			
4: Self-efficacy through skill development	• Trained facilitators leading activities • Programmes that foster skill development and confidence • Mentorship opportunities	• Feelings of self-efficacy • Empowerment and skill acquisition • Learning and teaching (service) practical skills	• Development of new skills • Improved self-esteem and confidence • Increased self-efficacy and empowerment
If programmes include trained facilitators and foster skill development and mentorship opportunities (context), then participants will feel empowered, acquire new skills, and experience increased self-efficacy (mechanism), because this improves self-esteem, confidence, and practical skills, fostering personal growth (outcome).			
Leadership, role modelling and belonging: Service			
5: Empathetic leadership	• Charismatic and empathetic leaders • Hierarchical structures • Service ethos	• Feelings of camaraderie • Sense of belonging • Place attachment • Feeling valued	• Improved social wellbeing • Stronger community bonds • Increased participant retention
If programmes are led by empathetic leaders in structured hierarchies with a service ethos (context), then participants will feel camaraderie, a sense of belonging, and place attachment (mechanism), because this enhances social wellbeing, strengthens community bonds, and increases participant retention (outcome).			
6: Intergenerational and peer mentoring	• Programmes involving various age groups/experience levels • Mentorship opportunities • Peer-led guidance	• Mutual learning and skill development • Sense of agency • Feeling useful	• Improved mental health for mentors and mentees • Enhanced confidence and self-esteem • Development of new skills • Stronger community bonds
If programmes integrate participants across age groups and experience levels, fostering peer mentoring and guidance (context), then participants will experience mutual learning, a sense of agency, and usefulness (mechanism), because this leads to improved mental health, self-esteem, and skill development for both mentors and mentees (outcome).			
Gendered, cultural, and community considerations: Growing together			
7: Male sensitivity and tailored programming	• Tailored male programming • Activities recognising and respecting cultural/background differences • Unique ways men engage through nature	• Feeling understood and respected • Feelings of unity • Identity formation	• Greater (cultural) inclusivity • Improved relations and respect among participants • Increased engagement and satisfaction
If programmes are tailored to male-specific needs and respect cultural differences (context), then participants will feel understood, respected, and develop a unified identity (mechanism), because this fosters greater inclusivity, improved interrelations, and increased engagement and satisfaction (outcome).			
8: Community building and support networks	• Creation of post-programme associations • Opportunities for continued engagement • Fostered social interaction	• Sense of belonging • Place attachment • Identity formation	• Long-term wellbeing maintenance • Continued health benefits post-programme • Sustained social support and community integration
If programmes create opportunities for post-programme social interaction and continued engagement (context), then participants will form strong support networks, develop place attachment, and experience identity formation (mechanism), because this ensures long-term wellbeing, sustained health benefits, and community integration beyond the programme (outcome).			

The initial step involved a rapid review of literature related to NBIs for men’s health and wellbeing. This initial scoping revealed limited examples of NBIs specifically targeting men, leading to an expanded scope that included interventions like Men’s Sheds, Walking Football, and Street Soccer, alongside wider public health and environmental health literature with a gender-specific focus. This broader approach enabled the identification of general themes and specific theories across various subdomains. This contributed to the formation of a provisional framework which was refined through ongoing discussions with two expert stakeholders – a male peer facilitator with lived experience of marginalisation, and a senior academic/registered social worker with practice experience in men’s health and NBIs. Both men brought lived and professional expertise in designing and delivering interventions with and for men. Their input was critical in refining the IPTs through interview-style consultations where they reviewed early frameworks derived from scoping work. A thematic synthesis of these discussions informed the refinement of the IPTs (see Supplementary File 1), enhancing the review’s validity. This process aligned with the rigour criterion outlined by Wong et al. [35].

### Testing the explanatory framework

The iterative literature search was completed on the 5th February 2024. Multiple electronic databases were searched: MEDLINE, PsycINFO, GreenFile, SocINDEX, CINAHL, Health Source, SPORTDiscus, Scopus, Social Care Online, Web of Science, Natural Science Collection, and Wiley Online Library, focusing on studies published in English from 2004 to 2024. Both free-text terms and subject headings were used to enhance search precision. The search string (see Table 2) included terms like ‘men’, ‘greenspace’, ‘nature therapy’, ‘forest bathing’, ‘horticultural therapy’, and others.

**Table 2:** Search string table

Population	
“men”, “males”	

Intervention	
greenspace, “green space”, “green care”, greencare, “nature therap*”, “wilderness therap*”, “outdoor behav* healthcare”, “outdoor behav* therap*”, “forest bathing”, “shinrin yoku”, “horticultural therap*”, “therapeutic horticulture”, “green exercise”, “ecotherapy”, “conservation therap*”, “care farming”, “men’s sheds”, “walking football”, “street soccer”	

Grey literature was identified through relevant websites, expert referrals, and informal networks (see Table 3). The search included various study designs, such as qualitative reports, randomised controlled trial (RCT) data, observational studies, and more.

**Table 3:** Grey literature table

Organisations included in grey literature search		
UK	Europe	International
Venture Trust	Asociacion Experientia	Street Soccer
Phoenix Futures		Men’s Sheds
The Wilderness Foundation		
Forest Therapy Scotland		
Cyrenians		
Venture Scotland		
The Green Team		
Way of Nature UK		
The Life Centre North		
Men2Men Woodland Therapy		
Street Soccer		
UK Men’s Sheds Association		
Walking football Scotland		
Both Sides Retreats		

### Selection and appraisal of documents

The search and selection process was conducted by one reviewer (JD). Duplicates were removed in Zotero and titles/abstracts were screened in Rayyan [49] for relevance and eligibility, with 20% of records blind-screened by a second reviewer (HC) for quality assurance. Discrepancies between reviewers were resolved by consensus to ensure consistent application of inclusion and exclusion criteria. At this stage, records were excluded if they were clearly: (i) not nature-based or greenspace-related interventions; (ii) not focused on adult humans; (iii) not about men’s health or did not report sex-disaggregated data; (iv) not empirical evaluations (e.g., editorials, commentaries, protocols, purely theoretical papers); or non-English. Full texts were then assessed against inclusion criteria guided by the research questions and IPTs, focusing on studies examining men’s participation in NBIs and contributing CMO-relevant data. During full-text screening and data extraction, exclusion criteria were iteratively refined to remove studies that did not focus on male participants, did not include a nature-based component, lacked usable outcome data, or provided insufficient detail to inform CMO configurations.

### Assessing relevance, richness, and rigour

Relevance, richness, and rigour were assessed throughout searching, extraction, and synthesis as these are fundamental to ensuring review quality and credibility. Relevance focused on the alignment of documents with the topic area and IPTs. Richness was evaluated based on the contribution of documents to theory building, with those concerning complex, multi-component NBIs for men receiving higher richness ratings. Simpler, often single activity interventions, like walking, were included when offering valuable insights. Rigour was assessed at both data and theory levels, considering the trustworthiness of sources based on their methodologies [50, 51]. Rigour was also assessed at the theory level, ensuring programme theories provided robust explanations of the available data. This process followed criteria for explanatory coherence, including simplicity and alignment with existing credible theories, such as the various middle-range theories (a theory that operates between general, abstract theories and specific, narrow hypotheses) highlighted in the findings and discussion sections, and explicated elsewhere [52–54]. Draft assessments and theory refinements were continuously discussed within the review team to challenge assumptions and reduce interpretive bias.

### Changes in the review process

During data extraction it became clear that, although highly relevant to men’s community health, the Men’s Shed studies did not always meet our a priori definition of a NBI. This is because many are primarily indoor or workshop-based, with nature peripheral rather than core. We therefore chose to use Men’s Sheds mainly to inform and test programme theories, rather than include them which would have dominated the NBI evidence base. Detailed CMO extraction was undertaken for 12 ‘Shed’ studies selected to maximise variation in country, setting, and population; these consistently reproduced the same CMO patterns. Additional Shed papers (*n* = 67) were retained as contextual/background material. They were not treated as primary data sources due to the risk that one quasi-NBI model would disproportionately shape the synthesis.

In parallel, IPTs were reformulated after extraction from approximately 25% of included papers (*n* = 10) to correct an initial bias towards early expert/scoping work on men with problem substance use. The revised IPT set expanded from 8 to 11 theories and subsequent full-dataset synthesis and refinement resulted in the 9 programme theories reported in the Findings.

### Data extraction

Data extraction focused on elements relevant to context, mechanisms and outcomes (CMOs) and on key descriptive characteristics of NBIs for men’s wellbeing. For each included study, the team recorded country, setting, target population, intervention type and components, study design, sample size and follow-up duration, alongside any available information on recruitment, and comparison conditions (where present). For outcomes, all reported health and wellbeing indicators for men (or sex-disaggregated male data) were extracted, including physical, mental, social and recovery-related measures at all available post-intervention time points. A pre-designed extraction form guided the collection of this information, including main findings and comments on CMO configurations (see Supplementary File 2). Each article’s alignment with the IPTs was recorded in an Excel matrix, noting how the study supported, refined or challenged the developing programme theories. To ensure consistency and rigour, 20% of included studies were independently cross-verified by a second reviewer (WM) using the same extraction framework. Extracted CMOs and IPT alignments were compared, minor interpretive differences were resolved by consensus, and no major discrepancies were identified; a third reviewer was therefore not required. This cross-checking helped minimise extraction bias and calibrate CMO interpretation across the team. Iterative searching continued alongside data extraction, and the search was considered complete once additional literature no longer contributed new CMO insights or refinements to the programme theories, consistent with realist principles of theoretical saturation.

### Data synthesis and analysis

The data synthesis strategy focused on elucidating the CMOCs that explain how NBIs facilitate men’s health and wellbeing. Data were first compiled into evidence tables categorising the context, mechanism, and outcomes reported in each study. Analysis of these tables detected patterns or demi-regularities (i.e., recurring patterns of outcomes or mechanisms that are observed across different contexts, but not universally), which assisted in crafting CMOCs. Through this analysis and identification of CMOCs, the IPTs were refined, with new programme theories also emerging to better explain observed outcomes.

### Findings

**Fig. 1:**
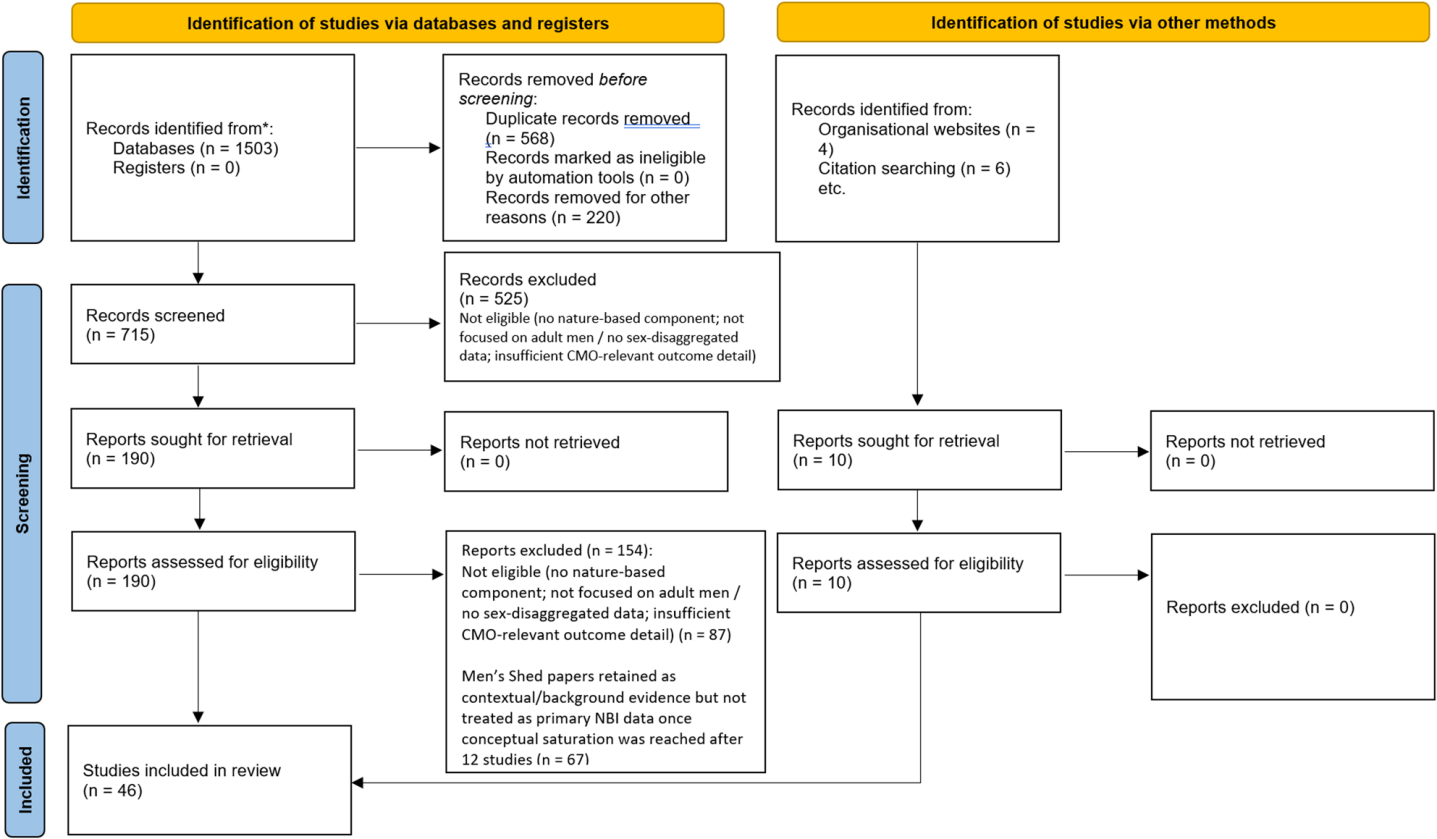
PRISMA (2020) [55] flow diagram

A total of 1,503 records were identified via database searches. After removing 568 duplicates and 220 records for relevance, 715 records proceeded to screening. Of these, 525 were excluded, leaving 190 reports sought for retrieval. All were successfully retrieved and assessed for eligibility, with 87 excluded for ineligibility and 67 for saturation. An additional 10 records were identified through other methods (4 from organisational websites and 6 from citation searching). All were retrieved, assessed for eligibility, and included in the final review. In total, 46 studies were included in the realist review as a result of close reading of the full texts. Literature searching and screening results are reported in Fig. 1 using PRISMA [55]. Information provided in each study about the programmes and participants varied, whilst key characteristics of all included studies were recorded (see Supplementary File 3). The nine refined programme theories, grouped into the ‘Being’, ‘Doing’ and ‘Growing together’ domains, are summarised in Table 4, immediately below.

**Table 4:** Programme theories of nature-based interventions for men

PT #	Title	‘If-then’ statement (CMOC)	Refinements of IPTs into PTs	Explanation and relation to other PTs
PT1	Being: restoration and mental resilience	If NBIs provide a calming environment with easy nature access and trained staff (C), encouraging sensory experiences (M resource) and reflection (M reasoning), then this can trigger participants’ parasympathetic nervous system, reducing stress and providing a cognitive and emotional reset (O).	Empirical testing revealed significant overlap between IPTs 1 and 2, leading to them being merged into PT1. This refinement emphasised the role of accessible, biodiverse settings, and solitude-supportive programming in NBIs. Such environments, combined with sensory engagement, activate a calming parasympathetic response and encourage reflection, resulting in cognitive and emotional resets, reduced stress, and improved wellbeing.	Simply being in a peaceful natural setting can physiologically reduce stress by activating relaxation responses. Sensory experiences and opportunities for reflection help participants reset emotionally and cognitively. This foundational context supports other programme theories focusing on ‘being’ in nature.
PT2	Doing: physical strength and active engagement	If physical activities offer tailored options in a relaxed atmosphere (C), and trained leaders promote group engagement (M resource), then it can support commitment and understanding of physical health benefits (M reasoning), leading to better physical fitness and sustained programme involvement (O).	PT2 broadens IPT3 by incorporating a relaxed atmosphere, trained leaders, and varied activity options, accommodating diverse participant needs. Mechanisms now focus on active engagement and group participation, fostering physical empowerment and interest in health. Outcomes expand to include enhanced fitness, mental health, and greater overall programme involvement.	Engaging in diverse physical activities within a supportive environment encourages men to become interested in their physical health, leading to improved fitness and sustained involvement in the programme. This theory relates to PT3 and PT4 by focusing on ‘doing’ through active engagement and skill development.
PT3	Creating meaning through purposeful doing	If men facing challenges engage in meaningful activities (C), within supportive environments (C), and enabling purposeful action and social engagement (M resource), then they feel a sense of accomplishment and renewed purpose (M reasoning), leading to personal growth and identity reformation (O).	PT3 emerged as a standalone theory due to the breadth of supporting data, highlighting purposeful ‘doing’ as central to identity reformation and wellbeing. Drawing from IPT3’s mechanisms of identity formation and reconnection with the masculine self, PT3 shows how meaningful engagement within supportive environments fosters agency, accomplishment, and renewed purpose, facilitating both physical empowerment and personal growth.	Engaging in meaningful, purposeful activities helps men find a sense of accomplishment and purpose, facilitating personal growth and a reformed identity. This builds on PT2 and complements PT4’s focus on skill development, centred on ‘doing’ meaningful activities.
PT4	Self-efficacy through skill development	If programmes support skill development in a supportive setting (C), with peer-led groups and capable staff (M resource), then individuals feel valuable and physically empowered (M reasoning), potentially enhancing self-esteem, self-efficacy, and confidence (O).	PT4 broadens IPT4 by shifting from facilitator-led skill development to a peer-supported, group-based approach. Retaining core elements like self-efficacy and empowerment, PT4 emphasises that a supportive, peer-led environment enhances feelings of being valuable, aligning with evidence that self-efficacy and confidence grow more effectively in group-driven contexts than in facilitator-focused settings alone.	Skill development in a supportive, peer-led environment helps individuals feel empowered and valuable, boosting their self-esteem and confidence. This complements PT3 and PT7. While PT4 focuses on physical empowerment through skills, PT7 emphasises mental empowerment through cognitive engagement. Both focus on ‘doing’ through skill acquisition.
PT5	Service: brotherhood and social bonds	If programmes focus on participants’ strengths, are inclusive (C), and encourage leadership opportunities (M resource), they can then foster a sense of agency, belonging, and unity of purpose (M reasoning), leading to improved social wellbeing and community bonds (O).	PT5 merges IPT5 and IPT6 into an inclusive approach that emphasises leadership and participants’ strengths. While IPT5 centred on empathetic leadership, and IPT6 on intergenerational mentoring, PT5 broadens these to foster agency and unity of purpose. This refined PT shows that social wellbeing and community bonds thrive when leadership and mentoring are inclusive, supporting mutual purpose and belonging across diverse participant groups.	Inclusive programmes that leverage participants’ strengths and offer leadership opportunities foster agency and belonging, strengthening social wellbeing and community ties. This theory relates to PT6 on community building; both focus on social aspects and ‘doing’ through engagement, enhancing social cohesion.
PT6	Growing together: community building and support networks	If community building is encouraged with sensitivity and stable support (C), fostering shared experiences (M resource), then it can lead to sustained social networks and involvement (M reasoning), potentially ensuring long-term support and community integration (O).	PT6 refines IPT8 by emphasising sensitive facilitation and stable support in community-building, fostering shared experiences that sustain social networks. While IPT8 focused on post-programme associations, PT6 includes ongoing interventions like Men’s Sheds, highlighting the value of long-term support and community integration beyond formal programme boundaries.	PT6 emphasises the importance of community building and stable support, for sustained social networks and community integration. This builds on PT5; both focus on social cohesion and ‘doing’ through community engagement, further strengthening community bonds and support systems.
PT7	Learning and problem-solving for personal recovery	If programmes focus on learning, development, and change (C) through programming promoting cognitive engagement and critical thinking (M resource), then they can empower participants to understand and solve problems (M reasoning), potentially enhancing self-efficacy, collaborative skills, and recovery outcomes (O).	PT7 emerged as a refined, residual theory capturing the cognitive aspects of ‘doing’ from PT4 on skill development, while also encompassing structured cognitive-behavioural therapy (CBT) components noted in veteran-focused NBIs.	Programmes promoting cognitive engagement and critical thinking empower participants, enhancing self-efficacy and collaborative skills, leading to better recovery outcomes. This complements PT4; while PT4 focuses on physical empowerment through skills, PT7 centres on mental empowerment through cognitive engagement. Both contribute to ‘doing’ through personal development.
PT8	Emotional support and resilience	If men are provided with structured psychological and emotional support (M resource), opportunities for peer connection and spiritual engagement (M resource) within a safe and non-judgemental environment (C), then they are more likely to experience emotional validation (M reasoning), increased self-awareness, and build emotional resilience to face life challenges (O).	PT8 emerged as a foundational context, illustrating that structured psychological and emotional support, alongside peer connections within safe spaces, fosters resilience and self-awareness. Aligned with nearly all articles, PT8 shows that these core support elements are essential for participants’ emotional validation and personal growth.	Providing emotional and psychological support within a safe environment enables men to build emotional resilience, increasing self-awareness and the ability to face life challenges. This foundational context underpins other programme theories, especially those focusing on personal growth and recovery, such as PT3, PT4, and PT7. It supports both ‘being’ and ‘doing’ by ensuring participants are emotionally equipped to engage fully in programmes.
PT9	Tailored support and multidisciplinary expertise	If a programme is designed with an explicit focus on its target population’s specific needs (C) and is managed by a multidisciplinary team with the appropriate expertise (M resource), then participants will feel adequately supported and committed to the programme’s goals (M reasoning), enabling focus on the therapeutic aims of the NBI and supporting wellbeing outcomes (O).	Refined from IPT7, PT9 highlights the importance of tailored, need-specific programming led by multidisciplinary teams. This foundational context emphasises that when men feel well-supported, commitment to programme goals strengthens, enhancing therapeutic outcomes and overall wellbeing.	Emphasises tailoring programmes to specific needs and managing them with appropriate expertise, leading to participants feeling supported and committed, enhancing wellbeing outcomes. This foundational context applies across all programme theories, ensuring programmes meet participants’ specific needs. It is crucial for both ‘being’ and ‘doing’, as it enhances the effectiveness of interventions by aligning them with participants’ backgrounds and needs.

*C* Context, *M resource *Mechanism resource, *M reasoning* Mechanism reasoning, *O* Outcome

### Foundational contextual programme theories

Although numbered PT8 and PT9 in line with the order of theory development, these two programme theories – emotional support and resilience (PT8) and tailored support with multidisciplinary expertise (PT9) – operate as foundational contexts that underpin and amplify the mechanisms articulated in PT1–PT7, shaping men’s engagement, recovery and longer-term wellbeing across settings. Across the programme theories, features such as emotional safety, peer connection, cultural and gender-sensitive tailoring, and appropriate expertise recur as visible, cross-cutting conditions rather than isolated mechanisms, suggesting they are necessary for NBIs to work effectively for diverse groups of men.

### Programme theory 8. Emotional support and resilience

PT8 proposes that if men are provided with structured psychological and emotional support (M resource), opportunities for peer connection and spiritual engagement (M resource) within a safe and non-judgemental environment (C), then they are more likely to experience emotional validation (M reasoning), increased self-awareness, and build emotional resilience to face life challenges (O). This pattern was evident in Men’s Sheds and community men’s programmes that explicitly addressed holistic or biopsychosocial wellbeing [25, 56–59], in NBIs for veterans and men with stress or chronic illness [29, 60, 61], and in culturally grounded programmes for Aboriginal and Torres Strait Islander men [62, 63]. Across these, safe spaces, peer support, and reflective or spiritual practices allowed men to share distress, make sense of life changes and develop new coping strategies, with reported reductions in anxiety, stress and low mood and enhanced emotional resilience.

### Programme theory 9. Tailored support and multidisciplinary expertise

PT9 proposes that if a programme is explicitly designed around the needs of a defined group of men and is supported by a team with appropriate expertise, participants feel adequately supported and committed to the programme’s goals, enabling focus on therapeutic aims and wellbeing outcomes. Strong support came from tailored Men’s Shed programmes [25, 57, 58, 63, 64], culturally adapted Men’s Sheds for Aboriginal men [25, 62, 65], and condition-specific NBIs such as Walking Football for men with diabetes or prostate cancer, Wildman for men with stress, and nature-based therapies for veterans [27, 29, 61, 66–69]. These interventions were co-designed or clinically tailored, and often delivered by multidisciplinary teams, which participants described as relevant, acceptable and fitting, supporting high adherence and sustained engagement.

### Programme theories 1–7

#### Programme theory 1. Being: restoration and mental resilience

‘Being’ in nature is important for men facing physical, psychological, and social challenges because it offers respite from pressure and an opportunity to exist in the present moment, countering the relentless cycle of ‘doing’ associated with masculine norms [70].

Refinements leading to PT1 suggest that if NBIs provide a calming environment with easy nature access and trained staff (C), with provision of sensory experiences (M resource) aiding reflection (M reasoning), it can trigger participants’ parasympathetic nervous system (M), reducing stress and providing a cognitive and emotional reset (O) [27, 29, 30, 41, 60, 61, 68, 69, 71–87]. NBIs facilitate this reset through sensory immersion and mindful practices in calming natural environments [27, 29, 30, 60, 61, 68, 69, 79, 84, 86]. This restorative state often precedes deeper cognitive engagement linked to recovery from a range of conditions [27, 29, 61, 68, 69], connecting PT1 to PT7.

Accessibility shapes engagement. Proximity to green and blue spaces supports participation [60, 88], whilst parks, botanical gardens, and urban forests are vital for individuals with limited mobility, particularly older men [63, 71, 79].Where access is constrained by urbanisation or disadvantage, facilitated transport enables men to reach nature-rich environments [27, 69, 75]. Cultural familiarity further supports engagement, as seen in the widespread uptake of shinrin-yoku (forest bathing) in Japan [79].

Natural environments consistently promote shifts from sympathetic to parasympathetic states, with evidence from forests, soundscapes, and other sensory exposures [79, 83, 89–91]. These calming states enhance reflection [29, 41, 85] and support cognitive recovery and emotional regulation [73, 80, 89]. Foundational contextual elements described in PT8 and PT9 were central: facilitators and multidisciplinary teams created safety, guided reflective practices (e.g., Qigong – a Chinese practice of coordinated movement, breathing, and meditation for health and balance), adapted activities and supported men across varied settings including prisons [29, 41, 60, 68, 69, 75, 76, 80]. In several interventions, researchers temporarily fulfilled these facilitator roles to ensure safety and fidelity [78, 79].

In the reviewed literature, being in nature provides powerful cognitive and emotional reset opportunities across diverse settings. Across programmes, men reported reduced anxiety, depression, confusion and fatigue, and increased vigour [92, 93]. In some multi-component NBIs, with veterans, restorative experiences also supported symptom reduction, including anxiety and anger, and prepared participants for subsequent therapeutic work [27, 29]. Collectively, these outcomes highlight the role that being in nature can play in reducing stress and fostering healthier thinking and feeling [72, 88].

These findings align clearly with Attention Restoration Theory (ART) [94], and Stress Reduction Theory (SRT) [95] which propose that calm, non-threatening natural environments promote cognitive recovery and reduce stress.

#### Programme theory 2. Doing: physical strength and active engagement

Refinements leading to PT2 reflect that, when physical activities offer tailored options in a relaxed atmosphere (C), and trained leaders promote group engagement (M resource), it can support commitment and understanding of physical health benefits (M reasoning), leading to better physical fitness and sustained programme involvement (O). Interventions, including Walking Football [66, 67, 96], conservation programmes [72], physical health-focused Men’s Sheds [58, 62, 65, 97], and multi-component NBIs [27, 61, 68, 69], foster awareness of health benefits, encourage long-term positive health behaviours, and promote wellbeing through structured, health-oriented group activities. A relaxed, supportive environment is crucial, particularly as masculine norms often deter men from help-seeking in traditional settings; non-threatening NBI spaces therefore provide accessible, stigma-free entry points [58, 62, 65, 88, 97]. Men’s Sheds use inclusive, pressure-free atmospheres to normalise participation in physical activity [58, 65], whilst the informality in veterans’ programmes encourages relaxation [61, 69]. Culturally sensitive environments for Aboriginal men and conservation volunteers likewise enhance comfort and willingness to engage [62, 72]. Being comfortable in the company of other men within natural settings further supports relaxation and commitment [78, 79, 83, 84, 93].

Tailored physical activity enhances engagement for men with differing needs. Walking Football taps into competitive motivation while accommodating older adults and men with chronic conditions such as prostate cancer [67, 96]. Low-impact, nature-based exercises – Qigong, walking and forest bathing – offer flexible options aligned with varying functional capacity [27, 68, 74, 78, 79, 84, 93]. As participants understand the physical health benefits, their commitment increases [58, 62, 65, 97]. Improvements in fitness and strength, reinforced by peer encouragement, help sustain healthy behaviours beyond programme participation [66–68, 96, 98, 99].

Trained leaders are essential resources for ensuring safety, adapting activities, and promoting ongoing participation. In Walking Football, professional supervision ensures appropriate progression and accessibility [67, 96], supporting confidence and reducing dropout [66]. Facilitators in NBIs guide mindfulness or exercise, helping participants relax and recognise health benefits [27, 79], whilst sensitive leadership fosters group cohesion and inclusivity, particularly for men facing social or health challenges [61, 69, 98].

Across studies, structured physical activity consistently improved men’s physical fitness. Walking Football enhanced balance, strength and cardiovascular health [67], as forest bathing reduced pulse rate, increased vigour and alleviated stress [79, 84, 89, 93]. Wilderness programmes improved physical health and reduced stress, and in some cases supported reductions in substance use [27, 61, 68]. Team-based activities, including football training, improved endurance, oxygen uptake, and body composition [98], with camaraderie supporting sustained involvement [66–68, 96, 98]. The Men’s Sheds literature highlights the health benefits of physical activity [58, 62, 65, 97], and investigation of NBIs reveals that forest bathing reduces stress, further enhancing positive physical outcomes and overall wellbeing [79, 84, 93]. Walking Football and Street Soccer maintain fitness through friendly competition and team camaraderie [66, 67, 96, 98]. Wilderness programmes also improve self-care and reduce substance use [61]. Low dropout rates were common, even under challenging conditions like lockdowns [27, 61, 66, 67].

Deci and Ryan’s [100] Self-Determination Theory (SDT) closely aligns with PT2, as structured physical activities in supportive settings satisfy needs for autonomy, competence and relatedness. Trained leaders enhance competence, group activity fosters social connection, and accessible activity options support autonomy – together reinforcing sustained engagement and positive behavioural change among men [58, 67, 68].

#### Programme theory 3. Creating meaning through purposeful ‘doing’

Empirical testing gave rise to PT3 which argues that if men facing challenges are offered structure within supportive environments (C), and engage in meaningful, communal activities (M resource), then they feel a sense of accomplishment and renewed purpose (M reasoning), leading to personal growth and identity reformation (O). By engaging in communal, practical activities rather than traditional therapies, NBIs offer pathways to personal growth, social connection and renewed purpose [56, 57, 70]. Structured environments are essential for men experiencing mental health challenges, social isolation, or major life transitions. Men’s Sheds provide predictability and belonging for older men adjusting to retirement [56–58, 63, 97], whilst care farming offers responsibility and routine that supports men in the criminal justice system to rebuild confidence and purpose [41, 73]. NBIs that incorporate ritual, physical practice or mindful engagement support clarity and stability for men with long-term illness or stress [27, 68, 101]. Structured sports programmes such as Walking Football and Street Soccer similarly create routine and socially engaging settings for older adults and men facing homelessness [96, 98].

For Aboriginal and Torres Strait Islander men, Men’s Sheds act as culturally safe, co-created spaces where purposeful activities are embedded in yarning, mentoring and shared illness stories, generating hope, belonging and renewed cultural identity, and thereby supporting personal growth and wellbeing [25, 62, 63].

Engagement in meaningful, communal activities works effectively by fulfilling men’s need for purposeful action and connection. Activities like woodworking and skill-sharing provide a sense of purpose, especially for older men navigating retirement or isolation [59, 64, 102]. Conservation volunteering offers men a way to engage in environmental stewardship, strengthening community ties, especially in urban settings where opportunities for connection are fewer [72]. NBIs integrating hands-on or mindful practices appeal to men with mental health needs, offering structured function-oriented activity [27, 68]. Across settings, purposeful doing yields shared achievement and integration within supportive groups.

Personal growth and identity reformation emerge as men recognise their capacity to learn, adapt and contribute meaningfully. In Men’s Sheds, participants often redefine their identities beyond work roles, taking on positions as mentors or leaders – an important transition for men experiencing post-retirement adjustment [56, 57, 59, 63, 64, 102]. Care farming and horticulture help men involved in the criminal justice system reimagine themselves as skilled and responsible, supporting reintegration and long-term desistance [41, 73]. Wilderness experiences offer reflective sessions that help veterans reassess priorities and rebuild identity post-service [61, 69]. Team sports similarly promote physical and psychological transformation, helping men view themselves as capable and socially engaged [67, 96, 98].

Frankl’s (1963) work [103] reinforces PT3, suggesting that purpose can be found even in adversity. NBIs provide structured, meaningful activities within supportive environments, enabling men to transform hardship into opportunities for self-transcendence and renewed purpose. Through communal engagement, participants channel difficulty into constructive pursuits, strengthening autonomy and reshaping identity.

#### Programme theory 4. Self-efficacy through skill development

PT4 posits that if programmes prioritise active engagement and group participation (C) within peer-led, skill-sharing communities with knowledgeable staff (M resource), then participants feel valuable and empowered (M reasoning), fostering self-efficacy and confidence (O). Refinement of this theory showed that supportive settings, peer leadership and competent staff cultivate holistic empowerment beyond technical skill acquisition [56, 58, 59, 63, 64, 102]. PT4 complements PT3’s focus on purposeful doing and PT7’s emphasis on cognitive engagement, enriching the model of self-development and social wellbeing.

Active engagement and group participation mitigate isolation and stagnation. In Men’s Sheds, involvement in meaningful, outward-facing projects and conservation volunteering enables men to feel valuable within their communities [56–59, 63, 64, 72, 102]. Through group-based kinaesthetic and reflective tasks, NBIs promote empowerment and feelings of mastery [27, 68, 75].Veterans, men experiencing homelessness, and those with criminal justice involvement similarly benefit from contributing time and skills in group-based contexts, with these activities offering meaningful roles [41, 61, 69, 73], and protective effects against suicide risk [104].

Across all settings, peer-led, skill-sharing communities, supported by knowledgeable staff, create structured but flexible environments for capability building. Men’s Sheds exemplify this through leadership opportunities in woodworking, gardening, and project management, with skilled peers modelling good practice [25, 64]. Staff in Sheds and Adventure Therapy programmes integrate experiential learning and health promotion to help men collaborate, model solutions and build problem-solving capacity [58, 75]. Walking Football provides tailored sessions balancing fitness and camaraderie, while nature-based interventions such as Qigong and storytelling draw on facilitators to guide safe, inclusive participation [27, 67, 68].

Across Indigenous-focused Men’s Shed literature, structured mentoring and culturally contextualised skill-building help Aboriginal and Torres Strait Islander men reclaim valued roles, develop new competencies and support other vulnerable groups, thereby enhancing self-efficacy and confidence [62, 63, 65].

Participation in such communities fosters empowerment and a sense of being valuable. Men report increased self-worth through visible contribution, leading Shed projects, improving physical capacity in sports, or engaging in conservation efforts [59, 66, 72]. Shared achievements reinforce capability and reshape identities [60, 76]. These effects are strengthened when cultural identity is validated and integrated into activities, promoting reciprocity, belonging and confidence [25, 62, 63, 65].

Self-efficacy and confidence are consistently fostered when men acquire and apply new skills in supportive, peer-led environments. Men’s Sheds, care farming, and conservation programmes enable technical skill development, the adoption of leadership roles, and personal health management [41, 64, 72]. Activities such as Walking Football and Street Soccer promote mastery over physical health, while nature-based and Adventure Therapies enhance problem-solving capacities and emotional regulation [61, 67, 75]. These experiences boost confidence as participants achieve personal and collective goals, feel valuable within their communities, and overcome stress through structured yet restorative environments [25, 68, 76].

Self-efficacy theory [105] notably aligns with PT4, providing theoretical understanding of the causal pathway. The central tenets of Self-Efficacy Theory, mastery experiences, vicarious learning, and social persuasion, map on to PT4. Success in skill-based tasks (mastery) builds self-efficacy and empowerment, while observing peers (vicarious learning) boosts confidence. Supportive feedback from facilitators (social persuasion) encourages perseverance, enhancing personal value and capability.

### Programme theory 5. Service: brotherhood and social bonds

Refinements leading to PT5 suggest that if programmes focus on participants’ strengths, are inclusive (C), and encourage leadership opportunities (M resource), they can then foster a sense of belonging, and unity of purpose (M reasoning), leading to improved social wellbeing and community bonds (O).

Strength-based and inclusive programmes create environments where men feel valued. Men’s Sheds engage older men, including those with fewer years of formal education, by drawing on skills in woodworking, repairs, conservation, sport and horticulture, activating belonging and shared purpose [56, 57, 63, 64, 102]. Inclusivity is enhanced by accommodating diverse backgrounds, including Indigenous and immigrant men, and those with disabilities, and addressing barriers like trauma, discrimination, and economic challenges through providing meals, transportation, and accessible, targeted activities [25, 58, 62, 68]. Male-only, judgement-free spaces promote comfort, fostering unity and belonging among participants [57, 59, 60, 63, 64, 72, 101, 102].

Leadership opportunities are central to PT5. Self-governance structures enable participants to lead activities and manage projects, supported by access to tools and collaborative spaces [25, 56, 58, 63, 64]. Peer-led health promotion positions men as decision-makers [58, 97], whilst intergenerational mentoring and conservation volunteering provide reciprocal learning roles [25, 65, 72, 88]. In Indigenous contexts, kinship structures, Elder-led mentoring, yarning and shared storytelling embed leadership within Aboriginal ‘men’s business’, generating trust, shared identity and emotional safety [25, 62, 65]. These culturally grounded roles help men reclaim cultural responsibilities and support other vulnerable groups, though some social norms may inadvertently reinforce unhealthy behaviours [63]. Nature-based programmes also embed leadership through group storytelling, rituals and exercises [27, 29].

Collaborative engagement strengthens belonging and unity of purpose [56, 59, 62, 63, 65]. Participants report feeling valued and integral to their groups, with their contributions perceived as vital to collective successes [57, 64]. Culturally tailored and inclusive programmes reinforce unity by recognising identity, lived experience and cultural obligations [62, 68]. Shared tasks and collective decision-making bolster group cohesion and a shared identity [58, 72]. NBIs can also shift men from hyper-independence to interdependence, cultivating empathy and collective responsibility [60]. Participation in group tasks consistently reduces isolation and enhances social wellbeing [29, 63, 68].

Strength-based programmes promote social cohesion by creating spaces where men feel purposeful and connected. Co-designed health activities, collaborative projects, and skill sharing strengthen bonds and promote collective health and community goals [56–58]. Intergenerational mentoring supports mutual respect and cultural continuity [23]. Nature-based and outdoor group activities reduce isolation and build collective purpose through storytelling, conservation and reciprocal leadership [27, 68, 72]. Arts-based and peer-led activities promotes interdependence, facilitating transitions from isolation to active engagement [60, 63, 101]. Across settings, structured, inclusive environments reliably enhance cohesion, unity of purpose and social wellbeing [29, 59].

PT5 aligns with Social Identity Theory [106] which proposes that belonging emerges through group membership. Strength-based, inclusive programmes with leadership roles cultivate a ‘brotherhood’ effect, reinforcing social identity, reducing isolation and promoting long-term social wellbeing.

### Programme theory 6. Growing together: community building and support networks

Refinements of PT6 assert that if a programme prioritises community building (C), through peer support and group activities (M resource), it can lead to feelings of belonging and enhanced self-worth (M reasoning), potentially ensuring sustained social networks and increased resilience (O). This framework recognises that even time-limited interventions can cultivate long-term benefits when they nurture strong social ties and encourage continued involvement.

Programmes that prioritise community building create inclusive and supportive environments where men can connect through shared experiences, countering social isolation and fostering belonging [27, 41, 57, 58, 61–63, 65–67, 76, 77, 85, 96]. Men’s Sheds exemplify this through ongoing, peer-led initiatives that offer regular opportunities for meaningful activity, and social interaction, reinforcing self-worth [58, 62, 64, 65, 102]. Street Soccer and Walking Football similarly consistent group engagement for men facing health, social or age-related challenges, encouraging integration and sustained physical health benefits [66, 67, 98]. In contrast, time-limited, professionally led NBIs rely on continued engagement after programme completion to maintain impact [27, 61].

Peer support and group activities are central mechanisms that enable deep social ties. In peer-led initiatives, activities are tailored by and for the community, enabling cultures of productivity, mutual support, and continuous learning [57, 58, 63, 101]. Participants engage in group projects and social events, fostering camaraderie and collective identity [59, 102]. Sports-based interventions sustain participation through their meeting regularly, offering routine, a peer-led ethos, the intrinsic appeal of the activities, through which men contribute to team development [88–90, 96]. NBIs using outdoor challenges and reflective discussion create similar cohesion but their reliance on professionals may pose sustainability challenges without integrated peer support [27, 61].

In Indigenous contexts, Sheds function as culturally grounded community hubs where kinship structures, intergenerational mentoring and yarning practices build trust, emotional safety and long-term relational bonds [25, 62, 63, 65]. These culturally embedded forms of community building strengthen belonging and social inclusion for Aboriginal and Torres Strait Islander men, though group norms require care to avoid reinforcing unhealthy behaviours [63].

Participation in peer-supported group activities enhances belonging and self-worth. Collaborative projects and shared achievements help men feel valued and connected, echoing PT5 but with emphasis on long-term social ties [58, 61, 68, 69, 102]. Sports programmes likewise create inclusive environments where participants feel belonging and increased self-worth [67, 96, 98]. Regular team activities and wilderness challenges reinforce self-care and extend benefits beyond formal interventions [27, 61, 66, 96, 98].

These programmes nurture sustained social networks and increase resilience, with participants across programmes continuing to support each other beyond programme settings [67, 68, 98, 102]. The ongoing, accessible peer support network helps men cope with challenges that arise and enhances their social wellbeing [65, 67, 98, 102]. Sports and veterans programmes similarly generate lasting ties, and thus, resilient social systems [27, 61, 66–68, 96].

Asset-Based Community Development (ABCD) Theory [107], aligns strongly with PT6: by mobilising existing community strengths – skills, relationships, cultural knowledge and peer leadership – programmes reinforce belonging and collective capacity.

### Programme theory 7. learning and problem-solving for personal recovery

Refined PT7 suggests that if programmes focus on learning, development, and change (C), by engaging participants in cognitive and critical thinking activities (M resource), then they empower individuals to understand and solve problems (M reasoning), leading to enhanced self-efficacy, collaborative skills, and positive recovery outcomes (O). Here, ‘recovery’ refers to improvements across mental and physical challenges, including stress, trauma, social exclusion, chronic illness and major life transitions.

Programmes prioritising learning, development, and change create structured and supportive environments that enhance cognition and critical thinking among participants [27, 29, 58, 61, 63, 69, 73]. Building on foundational contexts of safety and inclusivity (PT8–PT9), NBIs, Men’s Sheds, and Walking Football are resource-rich and activity-focused and thus contrast to traditional health and rehabilitation services. NBIs provide restorative outdoor experiences and guided practices which overtly engage participants in first conceptualising then solving problems [27, 29, 61, 69, 73]. Men’s Sheds reframe health, life and practical challenges as shared problems to be addressed collectively, reinforcing the communal meaning-making described in PT3 and the empowerment mechanisms in PT4 [57, 58, 63].

Cognitive and critical thinking activities are the central resource mechanism in PT7. In Men’s Sheds, collaborative projects, mentoring and skill-sharing demand planning analysis and continual learning [57, 58, 63]. NBIs incorporate structured challenges, experiential learning and mindful reflection to stimulate cognitive engagement [27, 29, 61]. Activities such as Qigong, narrative meditation and sensory exercises encourage reflection and applied problem-solving [27, 29, 61]. In prison horticulture programmes, gardening and plant care require sustained attention, strategic thinking and planning, supporting rehabilitation and reintegration [61, 73].

Through peer support and cognitive engagement, participants build confidence and self-efficacy. In Men’s Sheds, shared problem-solving enables men to model skills, exchange ideas and develop leadership – reinforcing PT4’s mechanisms of mastery and capability [57, 58, 63]. Veterans participating in NBIs report enhanced abilities to manage stress, navigate mental health challenges, and develop healthier coping mechanisms through reflective and analytical therapeutic activities [29, 69]. Even in restrictive environments, experiential learning challenges participants to apply cognitive skills to real-life contexts, strengthening resilience and readiness for transition [61, 73]. These processes align with PT5 and PT6, which highlight the importance of social identity and community support in sustaining recovery.

Prioritising learning, development and cognitive engagement fosters improvements across mental, emotional and social domains. In Men’s Sheds, participants report stronger social networks, improved cognitive health, and greater resilience, enabling recovery from isolation and exclusion [58, 63]. The continuous learning and collaborative environments of the settings covered under PT7 contribute to participants’ redefined sense of purpose and self-worth, central to men’s recovery from a range of conditions and circumstances [25, 99]. NBIs across participant groups report significant reductions in stress, anxiety, and depression, alongside enhanced connectedness and interdependent relationships [27, 29, 60, 61, 69, 101]. Participants describe increased confidence, improved coping, reduced isolation and stronger wellbeing, contributing to rehabilitation, more effective management of ongoing challenges, and recovery [27, 29, 60, 61, 69, 73, 101]. Integrating cognitive and critical thinking activities within supportive environments thus equips men with skills and confidence to effectively address personal and structural challenges [58, 63].

Recovery-oriented frameworks such as CHIME – Connectedness, Hope, Identity, Meaning and Empowerment [108] – align strongly with PT7, reflecting how cognitive engagement within supportive communities (PT1, PT3, PT4, PT5, PT6) enables men to rebuild identity, develop purpose and strengthen agency during recovery, from a range of conditions and circumstances.

### What does not work? challenges limiting the effectiveness of nature-based interventions for men’s wellbeing

Despite the promise of NBIs in enhancing men’s health, certain factors may limit their effectiveness when contexts, mechanisms, and outcomes are not properly aligned. Understanding these challenges is crucial for designing interventions that meet men’s unique needs.

A primary challenge arises when programmes do not provide a safe and non-judgemental environment (PT8). Without this foundational context, men may not feel comfortable engaging or expressing vulnerability, inhibiting mechanisms like emotional validation and self-awareness. For instance, if interventions are conducted in settings where participants feel judged or stigmatised, the intended outcomes of emotional resilience and cognitive reset may not materialise. While men often face societal pressures to appear strong, both physically and psychologically, without supportive contexts they are unlikely to participate fully, hindering personal growth. Another limitation occurs when programmes are not tailored to men’s specific needs (PT9). Generic interventions that overlook cultural, social, or individual factors and desires may fail to engage men effectively. Without addressing diverse needs through culturally sensitive and inclusive environments and programming, participants may not experience a sense of belonging or unity of purpose. This misalignment between context and mechanism can obstruct the development of social wellbeing and community bonds, which are vital for men’s health, highlighting the need for tailored emotional and psychological support within a safe environment (i.e., PT8 and PT9).

Interventions that do not offer meaningful activities aligned with men’s interests (PT3) may not foster purposeful action or social engagement. If programmes are not designed to resonate with men’s preferences, or do not provide opportunities for purposeful ‘doing’, men facing life transitions or challenges may not reconnect with a sense of identity or self-worth. This misalignment can result in missed opportunities for personal growth and identity reformation, as mechanisms of purposeful action and social engagement are not effectively activated. Importantly, programmes must acknowledge that not every man will prescribe purpose to the same activities, nor have the same interests. Assuming an understanding of ‘meaningful’ activity without consulting with individuals will likely hinder programme success. The absence of trained facilitators and capable staff also undermines NBIs’ effectiveness (PT1, PT4). Skilled leaders are essential for guiding activities, providing support, and fostering a supportive environment. Without them, mechanisms such as active engagement, skill development, and cognitive engagement may not be activated. For example, programmes lacking capable staff may fail to empower participants, leading to diminished self-esteem and confidence. Staff need both physical skills for nature-based work but also psychological and social skills to engage. For example, it appeared important for staff to be able to speak to shared experiences, to build rapport, and be personable. This sometimes requires a multidisciplinary team depending on the activity/therapeutic focus. The expertise of facilitators ensures that activities are meaningful and that participants feel valued and physically empowered.

Moreover, programmes that lack leadership opportunities or peer-led structures (PT5, PT6) may fail to enhance men’s self-esteem and sense of agency. Without mechanisms encouraging men to share skills, mentor others, or assume leadership roles, outcomes like strengthened community bonds and sustained social networks may not be achieved. Leadership roles and peer support are vital for fostering a sense of belonging and unity of purpose which are significant for men’s social wellbeing. Interventions that neglect cognitive engagement and critical thinking (PT7) may also be less effective. Men may benefit more when programmes promote learning, development, and change. Without these mechanisms, participants may not be empowered to understand and solve problems, limiting enhancements in self-efficacy and recovery outcomes. Programmes focusing solely on physical activities without integrating cognitive elements may likewise not fully engage men to make the link between their improved ability to solve problems in one domain and expanding this to addressing health and wellbeing challenges.

It is important to note that few studies explicitly report null or negative findings, and publication bias is likely. Much of the ‘what does not work’ narrative is therefore inferred from partial implementation, thin contextual description, or the absence of expected mechanisms, rather than from formal evaluations of failure. Taken together, however, the review suggests NBIs are least effective when emotional safety and tailoring (PT8, PT9) are weak; when activities lack meaning or agency (PT3, PT4, PT5, PT6); and when programmes overlook men’s need for cognitive engagement and learning (PT7). Designing interventions that attend explicitly to these conditions is therefore crucial for realising the potential of NBIs for men’s wellbeing.

## Discussion

This study identified several key mechanisms through which NBIs enhance men’s wellbeing. Natural spaces provide essential environments for reflection, facilitating cognitive reset and identity reformation. Physical activities tailored to men’s interests, such as team-based exercises, improve mood and foster camaraderie. Shared experiences within these settings build strong social bonds and reduce isolation, while supportive, peer-led facilitators enhance engagement and self-efficacy. Additionally, culturally relevant and gender-sensitive approaches are crucial for ensuring that NBIs effectively address the unique mental health needs of men, responding to recent calls (e.g., the Lancet Commission on gender and health equity) to bring men and boys more fully into gendered health agendas [19]. These findings highlight the importance of designing tailored, inclusive interventions that resonate with men’s personal and cultural identities to maximise the mental health benefits of NBIs. Taken together, and elaborated upon below, these programme theories address the research questions (RQ1-4) about how NBIs work to enhance men’s wellbeing, which contexts and mechanisms are most important, and how these may vary across different groups of men (RQ5).

This realist review contextualises these findings within persistent health disparities, noting men’s higher rates of premature death, externalising disorders, suicide, and substance use, which could themselves be considered gendered. By prioritising culturally relevant environments, structured peer-led activities, and supportive facilitation, the findings of this realist review highlight the need for a tailored approach to designing NBIs that cater specifically to men’s needs, enhancing the mental health benefits and overall impact of NBIs. Masterton et al.’s (2020) [38] realist review of greenspace for mental health provides a helpful framework, to which this current review adds new insights by incorporating men’s unique mental health needs and aligning with WHO’s [2] call for gender-responsive health systems. Thus, this exploration of NBIs as facilitators of men’s wellbeing aligns with, and contributes nuanced insights to, existing literatures [23, 38, 43, 109–112].

The findings across programme theories 1–7 demonstrate a robust alignment with and expansion upon the existing review-level literature on NBIs for health and wellbeing. Regarding PT1, the results corroborate the significant reductions in stress and anxiety reported by Lackey et al. (2021) [23] and Coventry et al. (2021) [43], emphasising the restorative impact of natural environments facilitated through sensory experiences and opportunities for reflection. Appeals to Attention Restoration Theory and Stress Reduction Theory are common across recent reviews, reinforcing the effect NBIs play in promoting a psychological reset [43, 110]. Similarly, PT2 is supported by evidence highlighting the effectiveness of active engagement in NBIs, such as hiking and group-based green exercise, in enhancing physical health and mood improvements [23, 43]. Present findings extend this by illustrating the importance of tailoring physical activities to men’s preferences, like Walking Football and conservation work, which not only improve fitness but also foster sustained commitment [67, 72, 96].

Furthermore, PT3 aligns with studies indicating that structured, meaningful activities within NBIs lead to personal growth and a sense of accomplishment [23, 43, 112]. The findings of this realist review expand upon this by highlighting how such purposeful engagement is particularly impactful for men by fostering identity reformation and empowerment, especially in contexts of transition into retirement and reintegration into civilian life [56–58, 61, 63, 64, 69, 70]. The findings in PT4 are consistent with the literature in demonstrating that skills-based activities enhance self-efficacy and confidence [38, 43, 111–113]. Additional insights emphasise the role of peer-led, skill-sharing communities in fostering empowerment among men [58, 65]. Moreover, PT5 and PT6 are reinforced by evidence showing that group-based NBIs foster social cohesion, reduce isolation, and enable the building of robust support networks [23, 43]. This study therefore adds depth to what is currently known by illustrating how strengths-based, inclusive programmes specifically enhance social bonds among men, promoting a sense of brotherhood and community integration [62, 102]. Multiple studies affirm PT7, demonstrating that cognitive engagement through structured activities and shared experiences in natural environments promotes mental recovery and enhances collaborative and problem-solving skills [43, 111]. These findings validate PT7’s emphasis on cognitive stimulation leading to mental health benefits.

The contribution notably expands the existing literature by addressing the significant gap in gender-specific research on NBIs, as highlighted by Lackey et al. (2021) [23] and Coventry et al. (2021) [43], who noted an overrepresentation of women and a lack of focus on gender differences. By integrating literature from men’s community health and wellbeing interventions, such as Men’s Sheds and Walking Football, with more traditional NBIs for men, this realist review provides a nuanced understanding of how NBIs can be tailored to their specific social and emotional needs [63, 96]. The importance of foundational contexts, such as safe, non-judgemental environments and programmes explicitly designed for men, is emphasised, enabling mechanisms across all programme theories to lead to positive health and wellbeing outcomes. This gender-sensitive approach demonstrates that when NBIs are adapted to align with traditional masculine values of action and purpose they are more effective in engaging men and fostering sustained benefits [70, 113, 114].

Whilst this realist synthesis necessarily sought to develop programme theories with broad applicability across diverse NBIs for men’s health and wellbeing, intersectional work in men’s health shows why NBIs cannot treat ‘men’ as a single group. Griffith (2012) argues that men’s health is shaped by ‘opportunity structures’ created by intersecting systems of race, class, place and gender, with Black men experiencing marked excess mortality yet remaining marginal in gender and health-equity infrastructures [115]. Smith (2020) similarly notes that global gender agendas largely sideline men and boys, masking inequities among men defined by Indigeneity, socioeconomic position and rurality, and calls for strengths-based, culturally tailored interventions grounded in social-determinants thinking [116]. Decolonising work with Aboriginal and Torres Strait Islander men shows how colonisation, racism, and dispossession intersect with gender, emphasising on-Country, kinship-based, and culturally grounded programmes as central to ‘thriving’ [117, 118]. In line with this, Men’s Shed studies in our review indicate that, for Aboriginal and Torres Strait Islander men, culturally safe, co-created Sheds that recognise kinship, Country and men’s business – and embed purposeful activities in yarning, intergenerational mentoring and strong service linkages – activate belonging, empowerment, emotional healing and sustained community support, refining PT3-PT6, PT8 and PT9 [25, 62, 63, 65]. Taken together, these literatures support the interpretation that NBIs for men must be theorised and designed with intersecting identities and structural conditions in view, rather than gender alone.

### Limitations

Several limitations should be considered when interpreting these findings. First, although the programme theories were developed to be broadly applicable, the evidence base is weighted towards marginalised groups of men (e.g., veterans, men with long-term conditions, men experiencing homelessness, Indigenous men in specific contexts) and towards economically advanced countries. Consequently, the theories are better supported for disadvantaged men in high-income settings than for men in the general population or in low- and middle-income countries.

Second, despite iterative searches and supplementary reading, the review under-represents both Indigenous/First Nations NBIs and intersectional men’s health interventions more broadly. The original strategy primarily combined generic male terms (e.g., men, males) with nature-based intervention terms and did not systematically incorporate intersecting identity terms (e.g., Indigenous, First Nations, Aboriginal, Torres Strait Islander, ethnicity, class, sexuality, disability, on-Country, land-based). As a result, some land-, sea- and on-Country programmes, and other culturally-grounded or intersectionally-framed NBIs, were not retrieved, and population group differences (RQ5) could only be explored where primary studies explicitly reported them. Intersectionality and decolonising scholarship, alongside Men’s Shed and veteran literatures, were used to interpret these differences, but the account of intersecting identities in men’s NBIs remains partial and indicative rather than comprehensive.

Third, heterogeneity and variable quality of primary studies constrains the strength of inferences. Many interventions were small, uncontrolled, short-term, and based on self-selected participants with limited follow-up, and the risk-of-bias appraisal indicated frequent issues such as selection bias, incomplete outcome data, and limited reporting. The published literature is also likely affected by publication bias, with positive or promising NBIs more often published than neutral or negative findings. Although some grey literature was included, publication bias could not be quantified, and the synthesis may over-represent successful programmes.

Finally, the realist approach and the authors’ positionality introduces interpretive elements. Decisions about relevance, rigour, and CMO configurations required judgement, and different reviewers might have configured the evidence differently. The lead author is a white man with lived experience of substance use, homelessness, and criminal justice involvement, which provides situated insight into some contexts discussed but may also shape what is foregrounded. The wider team are white Scottish/British women with backgrounds in social science/psychology, mental health/substance use/homelessness, and greenspace research. None of the wider team members have explicit lived experience of the issues in this review. All team members have significant experience in conducting realist reviews across a range of topics. Transparency and trustworthiness were sought through PROSPERO registration, adherence to established quality standards [35], explicit reporting of search and appraisal procedures, and detailed documentation of theory development, some subjectivity naturally remains and the programme theories should thus be read as plausible, theory-driven explanations, rather than definitive causal models.

## Conclusion

This novel and comprehensive realist review highlights the significant benefits of NBIs for men’s mental health. It builds on existing research by introducing a gender-specific perspective. NBIs offer essential environments for escape, reflection, and physical activity, which appear to be particularly effective when tailored to men’s cultural and personal identities. Peer-led roles and supportive leaders and facilitators play crucial roles in enhancing engagement and fostering social bonds. By prioritising culturally aligned and structured activities, NBIs could address men’s mental health challenges more appropriately and effectively than some traditional offerings. While these tailored approaches reinforce the foundational benefits of outdoor and group-based activity, they also ensure that interventions are inclusive and impactful. Future efforts should continue to refine and adapt NBIs to meet the evolving needs of men, promoting resilience, social cohesion, and overall wellbeing.

## Supplementary Information

Supplementary Material 1

Supplementary Material 2

Supplementary Material 3

Supplementary Material 4

## Data Availability

The datasets generated and analysed during this realist review consist of publicly available articles retrieved from academic databases and grey literature sources. Full details of the search strategy, inclusion criteria, selected studies, and data extraction processes are provided in the methods section and/or Supplementary materials. Further data supporting this review are available from the corresponding author upon reasonable request.
