# Supplementary material for: Realist review of nature-based interventions for men: understanding the contexts and mechanisms necessary for successful outcomes
Source: BMC Public Health. 2026 Mar 5;26:1199. doi: 10.1186/s12889-026-26867-7 (PMC13072547; doi:10.1186/s12889-026-26867-7)
Supplement: Supplementary file 1 — Supplementary Material 1 [file 12889_2026_26867_MOESM1_ESM.docx]

# Supplementary file 1: Thematic synthesis of consultations

To refine programme theories for this realist review, two 45-minute interviews were conducted with experts holding both lived and professional experience of nature-based interventions (NBIs) for men. Informed by initial programme theories (IPTs) shared in advance, these discussions explored the mechanisms and contexts underpinning men’s engagement with NBIs.

Key themes included the task-oriented nature of men’s interactions with nature, the therapeutic contrast between natural and urban environments, and the balance between social and solitary experiences. Mentorship, skill-sharing, and storytelling emerged as critical for fostering community bonds, personal growth, and inclusion.

These insights refined the IPTs and deepened understanding of how NBIs address men’s unique challenges, offering practical and theoretical contributions to their design and delivery.

**Men's experience in nature and evolutionary perspectives**:

Both consultations underline men's task-oriented approach to interacting with nature. This interaction extends beyond physical challenges, suggesting a deeper, potentially evolutionary connection to the natural world. The interplay between 'doing' and 'being' emerged as vital, but under articulated, with physical engagement often paving the way to mental and emotional connections with nature.

**Contrast with modern life and resilience**:

Contrasting natural environments with urban settings highlights nature's authenticity and therapeutic potential. The contrast from grey spaces to green spaces, was noted as playing a role in the NBI effectiveness. Tackling challenges in nature fosters resilience and personal development, offering relief from the pressures and artificiality of modern life.

**Physical and mental engagement**:

The synthesis highlights the dual advantages of nature engagement. Activities such as hiking, fishing, or learning survival skills not only provide mental health benefits by redirecting attention from stress to the present task but also lead to states of peace and attitude resets. Moreover, physical and mental ‘fitness’ are closely linked.

**Learning, teaching, and group dynamics**:

Skill development and mentorship were emphasised as key components of nature-based activities for improving wellbeing outcomes of men. The significance of group dynamics, including leadership, camaraderie, and competence-based hierarchies, were noted for creating a sense of belonging, achievement and inspiration. The discussion also addresses broader cultural and societal concerns, including the importance of father figures and healthy mentorship. These group/dyadic experiences lay the groundwork for individual growth and the exploration of solitary pursuits.

**Social vs. solo experiences**:

Achieving a balance between social and solitary experiences in nature were identified as crucial for wellbeing interventions aimed at men from a variety of backgrounds. While group activities facilitate skill development and relationship building, solitary experiences encourage self-reflection and personal challenges. This balance is proposed as a comprehensive approach to addressing the complexities of modern life and enhancing therapeutic outcomes.

**Role of storytelling and cultural reflections**:

Storytelling and the sharing of experiences were believed to play a role in developing a collective narrative and strengthening community bonds. One consultant described the dynamics of fishing as both a shared and individual experience which included joint travel to the location, with on-route planning and expectation setting. Once at the lake, each individual selects their own pitch, sufficiently distant from the other. The journey home, and time together subsequently, involves recounting the solitary fishing experience, whilst elaborations can be expected through repeated storytelling.

**Social inclusion, equality, and therapeutic aspects**:

The importance of ensuring social inclusion and equality in nature-based activities is underscored, particularly for men from various backgrounds. These activities provide therapeutic benefits by fostering a sense of peace, accomplishment, and connection, supporting recovery from addiction and mental health challenges.
