# Supplementary material for: Realist review of nature-based interventions for men: understanding the contexts and mechanisms necessary for successful outcomes
Source: BMC Public Health. 2026 Mar 5;26:1199. doi: 10.1186/s12889-026-26867-7 (PMC13072547; doi:10.1186/s12889-026-26867-7)
Supplement: Supplementary file 3 — Supplementary Material 3 [file 12889_2026_26867_MOESM3_ESM.docx]

| Authors | Date of Publication | Country | Title | Intervention | Available participant info | Health and wellbeing outcomes | Methods | IPT supported |
| --- | --- | --- | --- | --- | --- | --- | --- | --- |
| Ahl et al. | 2017 | Denmark | How the Men’s Shed idea travels to Scandinavia | Men’s Shed | 40 men (retired, though age range not provided) | Increased self-esteem and confidence, improved social wellbeing and community bonds, enhanced mental health | Qualitative methods, interviews, observations, thematic analysis | 3, 4, 5, 7, 8 |
| Barbagallo et al. | 2023 | Australia | Australian Men’s Sheds and their role in the health and wellbeing of men: A systematic review | Men’s Shed | Men and boys (variable description of numbers, age ranges within included articles) | Improved mental wellbeing, social skills, enhanced community integration | Systematic review, PRISMA guidelines, narrative synthesis, quality assessment using CASP tool | 3, 4, 5, 6, 7, 8 |
| Barbosa | 2020 | Portugal | Feasibility and safety of a Walking Football programme in middle-aged and older men with type 2 diabetes | Walking Football | 31 men, age 64.4 ± 4.5 years, with T2D | Improved physical fitness, high adherence and enjoyment, safe and effective exercise strategy | Quasi-experimental study, pre- and post-session health checks, adherence and enjoyment tracking, monitored by professionals | 3, 4, 5, 7, 8 |
| Campos-Uscanga | 2022 | Mexico | Running in natural spaces: Gender analysis of its relationship with emotional intelligence, psychological wellbeing, and physical activity | Running | 331 runners (55.3% women), aged 18-80 years, from 20 states | Higher psychological wellbeing and emotional intelligence in men, lower emotional intelligence but higher running frequency in women | Cross-sectional study, online questionnaires, gender-specific analysis, statistical tests (t-tests, chi-square, logistic regression) | 1, 2, 3, 4, 7 |
| Capela et al. | 2023 | Portugal | Effects of Walking Football on adherence, safety, quality of life and physical fitness in patients with prostate cancer: Findings from the PROSTATA_MOVE randomised controlled trial | Walking Football | 50 men with prostate cancer. | Improved cardiorespiratory fitness, muscle strength, balance, high adherence and enjoyment | Randomised controlled trial, pre- and post-intervention assessments (QoL, CRF, muscle strength, balance), statistical analysis | 3, 4, 5, 7, 8 |
| Carragher et al. | 2022 | Ireland, Australia | Shedding light: A qualitative study of women's view on Men's Sheds in Ireland and Australia | Men's Sheds | Narratives from 26 significant women of male Shedders in Ireland and Australia, recruited through Shed coordinators. | Enhanced companionship, social support, reduced stress for women, improved mental health for men. Adjustments to retirement and changes in traditional gender roles. | Narrative inquiry, thematic analysis following Polkinghorne's method: transcription, immersive review, coding, theme condensation, and data interpretation. | 3, 4, 5, 6, 7, 8 |
| Cavanagh et al. | 2020 | Australia | Culturally appropriate health solutions: Aboriginal men ‘thriving’ through activities in Men’s Sheds/groups | Men’s Sheds | Authors state over 150 men from 26 Indigenous men's groups, across urban, regional, and remote Australia | Improved health and wellbeing through culturally safe environments, increased confidence, health awareness, and vitality for learning | Qualitative case study, ‘gatherings’ and ‘yarning circles’ with men, observations, facilitated by Indigenous representatives | 3, 4, 5, 6, 7, 8 |
| Colley et al. | 2022 | Scotland | Who benefits from nature? A quantitative intersectional perspective on inequalities in contact with nature and the gender gap outdoors | Nature-based activities | 19,441 respondents from Scottish Household Survey | Highlighted the value of nature contact for health and wellbeing, identified disparities in access to natural environments | Cross-sectional data analysis, regression models, bivariate relationships, logistic regression, weighted analyses to correct sampling bias | 7 |
| Cordier and Wilson | 2014 | International | Mentoring at Men’s Sheds: an international survey about a community approach to health and wellbeing | Men’s Sheds | 324 Men’s Sheds were surveyed in Australia, 59 International sheds | Enhanced self-esteem, confidence, social inclusion, and health awareness | Survey with descriptive and inferential statistics, analysed using SPSS | 3, 4, 5, 6, 7, 8 |
| Currie et al. | 2016 | Scotland | Greenspace matters: exploring links between greenspace, gender, and wellbeing with conservation volunteers | Conservation activities | 13 Conservation volunteers (six women and seven men) in a deprived Scottish city | Multiple health and wellbeing benefits, improved social connections, equality in greenspace engagement | Mobile ethnography, go-along interviews, participant observation, thematic analysis with NVivo | 1, 2, 3, 4, 5, 6, 7, 8 |
| Duval and Kaplan | 2013 | USA | Exploring the benefits of outdoor experiences on veterans | Multi-day group-based outdoor recreation (backpacking, canoeing) | 98 male veterans. | Significant improvements in psychological wellbeing, social functioning, and life outlook. Enhanced activity engagement, particularly strong for those with severe ongoing health issues. | Repeated-measure design assessing psychological wellbeing, social functioning, life outlook, and activity engagement using validated scales and factor analysis to ensure construct validity. | 1, 2, 3, 4, 5, 7, 8 |
| Farrier et al. | 2017 | UK | Mental health and wellbeing benefits from a prisons horticultural programme | Horticultural programme in prisons | 897 prisoners (both male and female, aged 18-65) | Improved mental health and wellbeing, increased confidence, social interactions, skills acquisition, and potential for post-release employment | Mixed-method impact evaluation with Green Gym© questionnaires, WEMWBS, and BNIM interviews | 1, 2, 3, 4, 5, 6, 7, 8 |
| Geniole et al. | 2016 | Canada | Restoring land and mind: The benefits of an outdoor walk on mood are enhanced in a naturalised landfill area relative to its neighboring urban area | Walking through a naturalised landfill vs. walking through an adjacent urban area | 31 male participants, mean age 24.61 years. | Improved mood, decreased stress levels, and increased attentional control in natural settings. Enhanced benefits for those with higher nature connectedness. | Within-subject experimental design, pre-post measures of mood, arousal, attention control, and stress responses. Statistical analysis using paired t-tests, repeated-measure ANOVAs, and regression. | 1, 2, 3 |
| Gillis et al. | 2008 | USA | The effectiveness of project adventure’s behaviour management programmes for male offenders in residential treatment | Adventure-based behaviour management programme (BMtA), outdoor therapeutic camping (OTP), Youth Development Center (YDC) | Sample focused on male youth admitted from January 1995 to January 2001 (N = 2,115). | BMtA participants showed significantly lower re-arrest rates at one, two, and three years post-release compared to OTP and YDC participants. Improved behavioural outcomes. | Comparative analysis of re-arrest rates, mean months to re-arrest, and Kaplan-Meier survival functions to estimate the probability of re-arrest. Statistical analysis using chi-square analysis and ANOVA. | 1, 2, 3, 4, 5, 6, 7, 8 |
| Green | 2023 | UK | “No country for old men”: The lawnmower maintenance society | Informal sport (walking football), self-help wellbeing group | Not specified | Improved mental health and wellbeing, reduced isolation, positive feedback from participants, potential for broader application. | Narrative approach, feedback from participants, reflective commentary. | 3, 4, 5, 6, 7, 8 |
| Harper et al. | 2020 | UK | The physiological, physical, and biomechanical demands of Walking Football: Implications for exercise prescription and future research in older adults | Walking Football | 17 male participants (aged 66 ± 6 years) with at least one year of Walking Football experience. | Improved cardiovascular fitness, potential for improved bone health, social and mental wellbeing. | Observational study, measures of heart rate, blood lactate, accelerometry data, and rating of perceived exertion. Statistical analysis using paired sample t-tests and coefficient of variation for reliability. | 3, 4, 7, 8 |
| Høegmark et al. | 2021 | Denmark | The Wildman programme – Evaluating the implementability and effects of a nature-based intervention tailored for men with stress and long-term illnesses | Nature-based rehabilitation programme | 114 men with mental health problems and long-term illnesses | Improved quality of life, reduced stress, increased use of nature for restoration | Matched-control study, baseline, post-intervention, and 6-month follow-up assessments (WHOQOL-BREF, PSS, PRS), participant feedback | 1, 2, 3, 4, 5, 6, 7, 8 |
| Høegmark et al. | 2022 | Denmark | The Wildman programme – Experiences from a first implementation of a nature-based intervention designed for men with stress and chronic illnesses | Nature-based rehabilitation programme | 20 men aged 18-78 with stress and chronic illnesses | Reduced stress symptoms, enhanced quality of life, improved physical and psychological health | Quasi-experimental study, baseline and post-programme assessments (QoL, stress levels), participant feedback, thematic analysis | 1, 2, 3, 4, 5, 6, 7, 8 |
| Hondagneu-Sotelo | 2017 | USA | Place, nature and masculinity in immigrant integration: Latino immigrant men in inner-city parks and community gardens | Urban parks and community gardens | 27 interviews with Latino immigrant men, part of a broader team ethnography and interview study with 57 participants. | Improved social wellbeing, reduced feelings of marginalisation, enhanced sense of belonging and community integration. | Team ethnography, interviews, and data transcription and coding. | 1, 2, 3, 4, 5, 6, 7, 8 |
| Hoseinpoor Najjar et al. | 2018 | Iran | Horticulture therapy effects on memory and psychological symptoms of depressed male outpatients | Horticultural therapy | 30 male outpatients diagnosed with chronic depression. | Improved memory, reduced depression, anxiety, and stress. | Pretest-posttest control group design, Rey–Osterrieth Complex Figure Test (ROCF) and Depression Anxiety and Stress Scale (DASS-44) used for assessments, data analysed using SPSS-18 software. | 1, 2, 3, 4, 5, 7, 8 |
| Jahangir | 2019 | India | Perceived meaning of urban local parks and social wellbeing of elderly men: A qualitative study of Delhi and Kolkata | Urban local parks | 47 men (noted by authors as elderly, though age range not specified) from Kolkata and Delhi, regular visitors to local parks. | Improved social cohesion, reduced feelings of isolation, enhanced sense of wellbeing. | In-depth interviews and observations, digitally recorded, transcribed, and analysed using WeftQDA software. | 1, 2, 3, 7, 8 |
| Kang et al. | 2022 | South Korea | Pilot study on the physio-psychological effects of botanical gardens on the prefrontal cortex activity in an adult male group | Botanical gardens | 9 Korean adult males, average age 29.6 years, abstained from caffeine before the experiment. | Reduced oxyhemoglobin concentrations in the prefrontal cortex, improved psychological responses, and reduced physiological stress levels. | Near-Infrared Spectroscopy (NIRS), Profile of Mood State (POMS) and Perceived Restoration Scale (PRS), paired t-test and Wilcoxon signed-rank test. | 1, 2, 7 |
| Lee et al. | 2011 | Japan | Effect of forest bathing on physiological and psychological responses in young Japanese male subjects | Forest bathing | 12 young Japanese male university students, mean age 21.2 years, without past or current mental disorders, cardiovascular or allergic diseases. | Increased parasympathetic nervous activity, decreased sympathetic activity, reduced salivary cortisol levels, improved positive feelings, and reduced negative feelings. | Controlled three-day field experiment with alternating exposures to urban and forest environments, physiological and psychological measures, data analysis using paired t-tests and Wilcoxon signed rank tests. | 1, 2, 3, 7 |
| Li et al. | 2016 | Japan | Effects of Forest Bathing on Cardiovascular and Metabolic Parameters in Middle-Aged Males | Forest bathing | 19 male subjects aged 40-69 years (mean ± SD: 51.2 ± 8.8), not on antihypertensive medication but with varying levels of blood pressure. | Reduced pulse rate, increased vigour, decreased anxiety, fatigue, and confusion, increased serum adiponectin levels, no significant changes in blood pressure or metabolic parameters. | Controlled walks. Measuring blood pressure, pulse rate, mood states, and biological markers before, during, and after walks. | 1, 2, 3, 7 |
| Li et al. | 2022 | Japan | Effects of forest bathing (shinrin-yoku) on serotonin in serum, depressive symptoms and subjective sleep quality in middle-aged males | Forest bathing | 20 males aged 57.3 ± 8.4 years, participated in a 3-day trip to a forest park and an urban area. | Increased serum serotonin levels, improved sleep quality, reduced depressive symptoms, increased vigor, and reduced fatigue. | Repeated measures. Blood samples for serotonin and lactic acid, POMS test, and subjective sleep quality questionnaire. Data analysed using paired t-tests. | 1, 2, 3, 7 |
| Li et al. | 2013 | Japan | Effect of forest environments on psychological response evaluated by the POMS test | Forest environments | Three experiments: 12 males (37-55 years), 13 females (25-43 years), 16 males (36-77 years), 53 and 98 subjects, walked for two hours in forest and urban environments. | Increased vigor, decreased anxiety, depression, anger, and confusion in forest environments. | Walking. Repeated measures. POMS test conducted before, during, and after trips, results compared. | 1, 2, 3, 7 |
| Lloyd | 2022 | UK | Positive Futures: Health and Wellbeing Impacts for Veterans Struggling with Civilian Life 2019-2022 Final Report | Multi-component NBI | Veterans struggling with civilian life (96% male, age range not specified). | Improved mental wellbeing, reduced substance use, enhanced social interactions, and better personal development outcomes | Mixed-methods evaluation using health and wellbeing data, participant interviews, and data from Venture Trust’s data management system | 1, 2, 3, 4, 5, 6, 7, 8 |
| Markham | 2021 | Australia | The personal and social impact of Men’s Sheds: A realist investigation, review and synthesis | Men’s Sheds | Not clearly specified | Enhanced health and wellbeing, social inclusion, skill development, reduced loneliness | Realist inquiry framework, case studies, observations, interviews, document analysis, literature reviews | 3, 4, 5, 6, 7, 8 |
| McGrath et al. | 2022 | Ireland | Sheds for life: health and wellbeing outcomes of a tailored community-based health promotion initiative for Men’s Sheds in Ireland | Health promotion initiative in Men’s Sheds | 421 Men’s Sheds participants. | Improvements in subjective wellbeing, mental wellbeing, social capital, and healthy eating | Purposive sampling, questionnaires at baseline, 3, 6, and 12 months, descriptive and inferential testing | 3, 4 ,5, 7, 8, |
| Moran | 2019 | UK | Back to nature? Attention restoration theory and the restorative effects of nature contact in prison | Outdoor green spaces and nature images | Survey of 86 prisoners in a medium-security men's prison in the UK, response rate 8.6%. | Increased feelings of calm, ability to reflect, reduced stress. | Anonymous paper-based survey, adapted Perceived Restorativeness Scale (PRS), Wilcoxon signed-rank tests for quantitative data, coding of qualitative responses. | 1, 2, 7, 8 |
| Moylan et al. | 2013 | Australia | The Men’s Shed: Providing Biopsychosocial and Spiritual Support | Men’s Shed | 21 men, varying ages and occupations | Increased self-esteem and empowerment, reduced isolation, sense of belonging, improved mental health | Qualitative case study, participant observation, semi-structured in-depth interviews, thematic analysis | 3, 4, 5, 6, 7, 8 |
| Murray et al. | 2019 | England | Care farming: Rehabilitation or punishment? A qualitative exploration of the use of care farming within community orders | Care farming | Not specified | Improved social interactions, personal growth, new skills, sense of achievement, reduced social isolation. | Interviews conducted at care farms and probation offices, theoretical thematic analysis, development of a logic model for offender-specific care farming. | 1, 2, 3, 4, 5, 6, 7, 8 |
| Ochiai et al. | 2020 | Japan | Relaxing Effect Induced by Forest Sound in Patients with Gambling Disorder | Listening to forest sounds | 12 Japanese male gambling disorder (GD) patients, aged 19-58 years. | Decreased oxyhemoglobin levels in prefrontal cortices, improved mood states, increased feelings of comfort and relaxation. | Experiment with high-resolution forest and city sounds, heart rate variability (HRV), near-infrared spectroscopy (NIRS), modified semantic differential method, and Profiles of Mood States (POMS). | 1, 2, 7 |
| Park et al. | 2007 | Japan | Physiological Effects of Shinrin-yoku (Taking in the Atmosphere of the Forest)—Using Salivary Cortisol and Cerebral Activity as Indicators— | Shinrin-yoku (forest bathing) | 12 male college students, aged 22.8 ± 1.4 years. | Lower cerebral activity in the prefrontal area, lower salivary cortisol levels, indicating relaxation and stress reduction. | Walking. Repeated measurements of salivary cortisol and cerebral activity, data analysed using one-tailed t-tests and Wilcoxon signed-rank tests. | 1, 2, 3, 7 |
| Park et al. | 2009 | Japan | Physiological Effects of Forest Recreation in a Young Conifer Forest in Hinokage Town, Japan | Forest recreation | 12 male university students, ages 21.8 ± 0.8 years. | Lower pulse rate, diastolic blood pressure, and LF/(LF+HF) components of HRV, indicating relaxation | Physiological measurements (HRV, blood pressure, pulse rate) | 1, 2, 3, 7 |
| Petersen et al. | 2021 | Norway, Germany, New Zealand | How Does Being Solo in Nature Affect Wellbeing? Evidence from Norway, Germany and New Zealand | Solo experiences in nature | 40 participants (26 females, aged 19-64) | Enhanced wellbeing across PERMA-V dimensions, minor variations by nationality, gender, age, prior experience, and expectations | Qualitative content analysis (QCA) of solo debrief responses | 1, 2, 3, 4, 5, 7 |
| Poulsen et al. | 2016 | Denmark | ‘Everything just seems much more right in nature’: How veterans with post-traumatic stress disorder experience nature-based activities in a forest therapy garden | Nature-based therapy for veterans | 8 male veterans with PTSD symptoms | Improved PTSD symptoms, increased self-efficacy, deeper relationship with nature | Qualitative study, interpretative phenomenological analysis, semi-structured interviews at multiple points (baseline, 5 weeks, 10 weeks, 1 year post-treatment) | 1, 2, 3, 4, 5, 6, 7, 8 |
| Randers et al. | 2012 | Denmark | Short-term Street Soccer Improves Fitness and Cardiovascular Health Status of Homeless Men | Street Soccer training | 55 homeless men (divided into training and control groups) | Improvements in VO2max, fat percentage, LDL cholesterol, and cardiovascular health profile | Training intervention with physical fitness and cardiovascular health assessments | 3, 4, 5, 7, 8 |
| Reynolds et al. | 2013 | Canada | The experiences of older male adults throughout their involvement in a community programme for men | Men’s Sheds | Older male adults, varied backgrounds | Enhanced social engagement, active aging, improved wellbeing, sense of purpose | Grounded theory, interviews, purposive and theoretical sampling, coding for data analysis | 3, 4, 5, 6, 7, 8 |
| Sassaman | 2022 | USA | Anchoring Masculinity in Interdependence through Outdoor Experiential Education | Nature-based mindfulness retreat | Teen boys in a week-long retreat, geographically representative sample | Reduced symptoms of depression, anxiety, stress, enhanced nature connectedness, and interdependent masculinity | Mixed methods, AIMS validation, case study, surveys, and interviews | 1, 2, 3, 4, 5, 6, 7, 8 |
| Seaman et al. | 2020 | Australia | Reaching at-risk rural men: An evaluation of a health promotion activity targeting men at a large agricultural event | Health promotion tent | 401 men, median age 56, from rural areas | Higher health awareness, identification of health risks, increased engagement in health monitoring | Cross-sectional study, health assessments, surveys, comparison with national data, statistical analysis | 3, 5, 7, 8 |
| Song et al. | 2013 (a) | Japan | Physiological and psychological effects of walking on young males in urban parks in winter | Walking in urban parks | 13 male university students aged 22.5 ± 3.1 years | Lower heart rates, increased parasympathetic activity, improved mood, decreased anxiety | Field experiment, physiological measurements (heart rate, HRV), psychological assessments (SD method, POMS, STAI) | 1, 2, 3 |
| Song et al. | 2013 (b) | Japan | Individual Differences in the Physiological Effects of Forest Therapy Based on Type A and Type B Behaviour Patterns | Forest therapy | 485 male university students, ages 21.8 ± 1.6 years | Type B individuals exhibited lower pulse rate and diastolic blood pressure in forest environments | Physiological measurements (pulse rate, blood pressure) and behaviour pattern classification | 1, 2, 7 |
| Song et al. | 2017 | Japan | Effects of Viewing Forest Landscape on Middle-Aged Hypertensive Men | Viewing forest landscapes | 20 hypertensive men, ages 58.0 ± 10.6 years | Increased parasympathetic nervous activity, decreased heart rate, and improved psychological responses | Physiological measurements (HRV, heart rate) and psychological assessments using the modified semantic differential method | 1, 2, 7 |
| Waling et al. | 2016 | Australia | Don't fix what ain't broke': evaluating the effectiveness of a Men's Shed in inner regional Australia | Men’s Shed | 22 surveys, 20 interviews with participants | High satisfaction with programme format, improved emotional wellbeing, enhanced social support, positive impact on interpersonal relationships | Community-level needs assessment, surveys, semi-structured interviews, community needs analysis framework | 3, 4, 5, 7, 8 |
| Way of Nature | N/A | N/A | Men's programme | Structured nature-based retreat | Not specified | Increased calm, happiness, self-awareness, reduced mental clutter, improved practices, sense of connection, realignment with personal values | Structured unstructured time, peer group support, personal development techniques, experiential learning, natural environment, and unplugged experience | 1, 2, 3, 4, 5, 6, 7, 8 |
| Will | 2016 | USA | Wilderness recreation - An Analysis of Social Carrying Capacity, Regional Differences, and the Role of Gender | Wilderness recreation | 2,559 visitors to wilderness areas in California and Oregon | High satisfaction levels, minimal crowding and conflict, significant gender-based differences in motivations and experiences | On-site surveys, statistical analysis (frequencies, ANOVA, t-tests, Chi square), regional and gender comparisons | 1, 2, 3, 7, 8 |
